# Traumatic Brain Injury Patients Mortality and Serum Total Antioxidant Capacity

**DOI:** 10.3390/brainsci10020110

**Published:** 2020-02-18

**Authors:** Leonardo Lorente, María M. Martín, Antonia Pérez-Cejas, Agustín F. González-Rivero, Pedro Abreu-González, Luis Ramos, Mónica Argueso, Jordi Solé-Violán, Juan J. Cáceres, Alejandro Jiménez, Victor García-Marín

**Affiliations:** 1 Intensive Care Unit, Hospital Universitario de Canarias, 38320 Ofra s/n. La Laguna, Spain; 2 Intensive Care Unit, Hospital Universitario Nuestra Señora de Candelaria, 38020 Crta del Rosario s/n., Spain; mar.martinvelasco@gmail.com; 3 Laboratory Department, Hospital Universitario de Canarias, 38320 Ofra s/n. La Laguna, Spain; aperezcejas@gmail.com (A.P.-C.); agonriv@hotmail.com (A.F.G.-R.); 4 Department of Physiology, Faculty of Medicine, University of the La Laguna, 38320 Ofra s/n. La Laguna, Spain; pabreu@ull.es; 5 Intensive Care Unit, Hospital General La Palma, 38713 Buenavista de Arriba s/n., Spain; lramosgomez@gmail.com; 6 Intensive Care Unit, Hospital Clínico Universitario de Valencia, Avda, Blasco Ibáñez nº17-19, 46004 Valencia, Spain; moni_begasa@hotmail.com; 7 Intensive Care Unit, Hospital Universitario Dr. Negrín, CIBERES, 35010 Barranco de la Ballena s/n. Las Palmas de Gran Canaria, Spain; jsolvio@gobiernodecanarias.org; 8 Intensive Care Unit, Hospital Insular. Plaza Dr. Pasteur, 35016 s/n. Las Palmas de Gran Canaria, Spain; juanjose.caceresagra@gobiernodecanarias.org; 9 Research Unit, Hospital Universitario de Canarias, 38320 Ofra s/n. La Laguna, Spain; ajimenezsosa@gmail.com; 10 Department of Neurosurgery, Hospital Universitario de Canarias, 38320 Ofra s/n. La Laguna, Spain; vicgarmar666@gmail.com

**Keywords:** total antioxidant capacity, traumatic brain injury, patients, mortality, prognosis

## Abstract

Objective: Oxidation is involved in secondary brain injury after traumatic brain injury (TBI). Increased concentrations of total antioxidant capacity (TAC) in blood at the time of admission for TBI have been found in non-surviving patients. The main objective of this study was to determine the role of serum TAC levels at any time during the first week of TBI for the prediction of early mortality. Methods: Isolated (<10 points in non-cranial aspects of Injury Severity Score) and severe (<9 points in Glasgow Coma Scale) TBI patients were included. Serum TAC concentrations at days 1, 4, and 8 of TBI were determined. The end-point study was 30-day mortality. Results: Higher serum TAC levels at days 1 (*p* < 0.001), 4 (*p* < 0.001), and 8 (*p* = 0.002) of TBI were found in non-surviving (*n* = 34) than in surviving patients (*n* = 90). The area under curve (95% Confidence Interval) for prediction of 30-day mortality by serum TAC concentrations at days 1, 4, and 8 of TBI were 0.79 (0.71–0.86; *p* < 0.001), 0.87 (0.79–0.93; *p* < 0.001), and 0.76 (0.67–0.84; *p* = 0.006) respectively. Conclusions: The novelty of our study was the ability to predict 30-day mortality by serum TAC concentrations at any time during the first week of TBI.

## 1. Introduction

Many disabilities and deaths occur due to traumatic brain injury (TBI) [1]. Oxidation is implicated in secondary brain injury after TBI [2,3,4,5]. Reactive oxygen species (ROS) produce secondary brain injury and are balanced by the action of antioxidant brain defenses [2,3,4,5]. Measurement of total antioxidant capacity (TAC) has been used to approximate antioxidant status better that measuring concentrations of each antioxidant compound [6]. 

There is little data on blood TAC levels in patients with TBI [7,8,9]. Previously, in one study with 44 TBI patients, higher serum TAC levels were found at 24 h and 48 h after TBI in patients with poor functional outcome at 6 months; however, the authors found no differences in serum TAC levels on admission between patients with poor and good functional outcome and no data on oxidative status were reported [7]. In another study with 54 TBI patients, higher serum levels of TAC and total oxidant status were found on admission in non-surviving patients; however, the authors did not report these serum levels during follow-up [8]. In an earlier study conducted by our team with 100 TBI patients, higher serum levels of TAC and malondialdehyde were found on admission in non-surviving patients; however, these blood levels were not reported during follow-up [9]. Malondialdehyde is an end-product of lipid peroxidation that appears in the blood and has been used to estimate lipid oxidation [10,11]. Therefore, the objectives of the study were to analyze serum TAC levels during the first week of TBI in surviving and non-surviving patients, to stablish whether there is an association between serum TAC levels and lipid peroxidation during the first week of TBI, and to analyze whether serum TAC levels during the first week of TBI can be used to predict early mortality.

## 2. Methods

### 2.1. Design and Subjects

Six Spanish hospitals participated in this prospective and observational study. The Institutional Board of each hospital approved the study: H. Universitario Dr. Negrín (Las Palmas de Gran Canaria), H. General de La Palma, H. Insular (Las Palmas de Gran Canaria), H. Universitario de Canarias (La Laguna, Tenerife), H. Universitario Nuestra Señora de Candelaria (Santa Cruz de Tenerife), and H. Clínico Universitario de Valencia. The written and signed consent for the participation in the study was obtained by a relative of each patient.

Patients with an isolated and severe TBI were included. Isolated TBI was considered when the patient had <10 points in the non-cranial aspects of the Injury Severity Score (ISS) [12]. Severe TBI was considered when the patient scored <9 points on the Glasgow Coma Scale (GCS) [13]. Patients with comfort measures only, inflammatory disease, age under 18 years, and malignant disease were excluded.

Sex, age, GCS, ISS, Acute Physiology and Chronic Health Evaluation II (APACHE II) score [14], activated partial thromboplastin time (aPTT), fibrinogen, international normalized ratio (INR), glycemia, bilirubin, lactic acid, creatinine, sodium, fraction inspired of oxygen (FIO_2_), pressure of arterial oxygen (PaO_2_), platelets, leukocytes, hemoglobin, brain lesions using Marshall computer tomography classification (CT) [15], cerebral perfusion pressure (CPP), and intracranial pressure (ICP) were recorded. The end-point study was 30-day mortality.

### 2.2. Serum Samples Collection 

Serum samples were collected on days 1, 4, and 8 of TBI and the samples were frozen at −80 °C until the serum concentration determinations.

### 2.3. Determinations of Serum TAC Levels 

Earlier, serum TAC concentrations were determined in one hundred patients on day 1 of TBI [9]. The inclusion of patients continued, and 124 patients (the first 100 patients and other 24 patients) had serum malondialdehyde concentrations determined (to assess lipid peroxidation) at days 1, 4, and 8 of TBI [16]. For these 124 patients with TBI, serum TAC concentrations on days 1, 4, and 8 were determined to establish their prognostic ability for early mortality and their association with serum malondialdehyde levels. The antioxidant assay kit (Cayman Chemical Corporation, Ann Arbor, MI, USA) was used for the determination of TAC in the Laboratory Department of the Hospital Universitario de Canarias from La Laguna, (Tenerife, Spain). The detection limit of this kit was of 0.04 mmol/L, and the intra and inter-assay coefficients of variation (CV) were of 3.4% and 3.0%, respectively. 

### 2.4. Determination of Serum Malondialdehyde Levels

For the determination of malondialdehyde levels at the University of La Laguna (Tenerife, Spain) the method of thiobarbituric acid reactive substances (TBARS) of Kikugawa et al. [17] was used. The assay detection limit of the assay was of 0.079 nmol/mL, and the inter- and intra-assay CV were 4.01% and 1.82%, respectively. 

### 2.5. Statistical Methods

Medians (75th and 25th percentiles) and frequencies (percentages) were used to report continuous and categorical variables. The Wilcoxon–Mann–Whitney and the chi-square test were used to compare continuous and categorical variables between surviving and non-surviving patients at 30 days. Receiver operating characteristic (ROC) analyses were used to test the prediction ability of 30-day mortality by serum TAC levels at day 1, 4, and 8 of TBI. In addition, specificity, sensitivity, negative predicted values and likelihood ratios, positive predicted values, and likelihood ratios for the cut-offs of serum TAC levels were reported at days 1, 4, and 8 of TBI (all cut-offs selected according to the Youden J index). The programs LogXact 4.1 (Cytel Co., Cambridge, MA, USA), SPSS 17.0 (SPSS Inc., Chicago, IL, USA), and NCSS 2000 (Kaysville, UT, USA) were used for statistical analyses. A statistically significant difference was considered with a cut-off value of *p* < 0.05. 

## 3. Results

**Figure 1:**
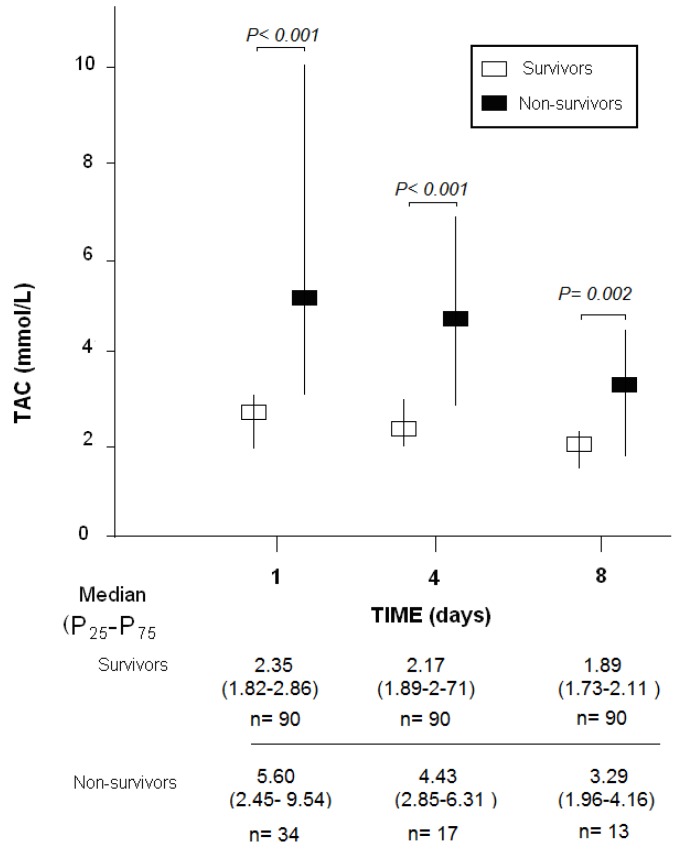
Serum total antioxidant capacity (TAC) levels at day 1, 4, and 8 of traumatic brain injury in 30-day surviving and non-surviving patients.

Survivors (*n* = 90) in comparison to non-surviving patients (*n* = 34) had higher GCS, lower APACHE-II score, lower age and a lower proportion of women (Table 1). There were also differences between surviving and non-surviving patients in the brain computer tomography findings. In addition, survivors had lower serum TAC levels at days 1 (*p* < 0.001), 4 (*p* < 0.001), and 8 (*p* = 0.002) of TBI than non-surviving patients (Figure 1). 

The area under curve (95% Confidence Interval) for predicting mortality at 30-day by serum TAC concentrations at days 1, 4, and 8 of TBI were 0.79 (0.71–0.86; *p* < 0.001), 0.87 (0.79–0.93; *p* < 0.001), and 0.76 (0.67–0.84; *p* = 0.006) respectively. Table 2 shows specificity, sensitivity, negative predicted values and likelihood ratios, and positive predicted values and likelihood ratios for the cut-offs of serum TAC levels at days 1, 4, and 8 of TBI. 

Lower serum malondialdehyde levels were found in surviving patients than in non-surviving patients on day 1 (1.35 (1.05–1.77) vs. 2.03 (1.36–4.12) nmol/mL; *p* < 0.001)), day 4 (1.12 (0.93–1.38) vs. 2.12 (1.63–2.36) nmol/mL; *p* < 0.001), and day 8 (1.07 (0.90–1.43) vs. 2.13 (1.98–2.28) nmol/mL; *p* < 0.001) of TBI. 

There was a positive association between serum concentrations of TAC and malondialdehyde on days 1 (rho = 0.25; *p* = 0.01), 4 (rho = 0.43; *p* < 0.001), and 8 (rho = 0.25; *p* = 0.01) of TBI.

## 4. Discussion

Previously, a study found higher serum TAC levels 24 h and 48 h after TBI in patients with poor functional outcome after 6 months; however, no data on the oxidant state was reported [7]. In another study higher serum levels of TAC and total oxidant status were found on admission in non-surviving patients [8]. In a previous study conducted by our team, higher serum levels of TAC and malondialdehyde were found on admission in patients who did not survive [9]. Therefore, the novel aspects of our current study were that we reported data on serum levels of TAC and malondialdehyde also on days 4 and 8 after TBI. In addition, the novelties of our study were the presence of higher serum TAC levels during the first week of TBI in non-surviving than in surviving patients, the positive association between serum levels of TAC and malondialdehyde during the first week of TBI, and the ability of serum TAC levels to predict mortality at 30 days of TBI at any moment of the first week of TBI. 

We have recollected several demographic and clinical variables that could be different between surviving and non-surviving patients. In addition to serum levels of TAC and malondialdehyde, other variables were statistically different between surviving and non-surviving patients at day 1 of TBI as GCS, sex, age, CT brain findings, and APACHE-II score.

We believe that those higher serum TAC concentrations during the first week of TBI in non- surviving patients and the positive association with serum malondialdehyde concentrations may be motived by an attempt to reduce the high ROS production and lipid peroxidation in non-surviving patients. However, unfortunately those higher serum TAC concentrations in non-surviving patients are not enough to compensate the unfavorable clinical situation and the patient eventually dies.

Our study has the limitation that data on other compounds of oxidant and antioxidant states were not reported. However, we believe that the results of our study and the beneficial effects found with the antioxidant agents administration in TBI animal models (reducing oxidative status and neurological deficits) [18,19,20,21,22,23,24,25] could motivate the research on TAC in TBI patients. We believe that validating the role of serum TAC concentrations at any time during the first week of TBI could be interesting in the prediction of mortality in TBI patients because it could help clinicians in prognosticating these patients. In addition, research into the administration of antioxidant agents in TBI patients to reduce oxidative status and neurological deficits may be of interest.

## 5. Conclusions

The novelty of our study was the ability to predict 30-day mortality by serum TAC concentrations at any time during the first week of TBI.

## Figures and Tables

**Table 1:** Characteristics on admission of 30-day surviving and non-surviving patients.

	Non-Surviving (*n* = 34)	Surviving (*n* = 90)	*p*-Value
GCS-m (p 25–75)	4 (3–7)	7 (5–8)	<0.001
Gender female-n (%)	13 (38.2)	15 (16.7)	0.02
Age (years)-m (p 25–75)	65 (55–75)	46 (28–62)	<0.001
ISS-m (p 25–75)	25 (25–25)	25 (25–34)	0.28
Marshall computer tomography-n (%)			0.01
Diffuse injury I	0	0	
Diffuse injury II	5 (14.7)	25 (27.8)	
Diffuse injury III	6 (17.6)	15 (16.7)	
Diffuse injury IV	9 (26.5)	13 (14.4)	
Evacuated mass lesion V	6 (17.6)	32 (35.6)	
Non-evacuated mass lesion VI	8 (23.5)	5 (5.6)	
PaO_2_ (mmHg)-m (p 25–75)	142 (97–195)	148 (110–242)	0.45
PaO_2_/FIO_2_ ratio-m (p 25–75)	294 (167–395)	336 (246–400)	0.11
Platelets-m*10^3^/mm^3^ (p 25–75)	172 (125–232)	182 (135–238)	0.49
aPTT (seconds)-m (p 25–75)	29 (25–37)	28 (25–31)	0.25
Fibrinogen (mg/dl)-m (p 25–75)	348 (300–475)	371 (286–471)	0.70
INR-m (p 25–75)	1.12 (1.03–1.48)	1.11 (1.00–1.24)	0.19
Leukocytes-m*10^3^/mm^3^ (p 25–75)	14.9 (9.7–21.6)	13.9 (10.1–19.0)	0.47
Hemoglobin (g/dL)-m (p 25–75)	11.9 (10.0–13.7)	11.2 (10.0–13.0)	0.73
Bilirubin (mg/dl)-m (p 25–75)	0.70 (0.53–1.05)	0.60 (0.40–0.80)	0.06
Glycemia (g/dL)-m (p 25–75)	160 (125–191)	139 (121–167)	0.11
Sodium (mEq/L)- m (p 25–75)	141 (136–147)	140 (138–143)	0.41
Creatinine (mg/dl)-m (p 25–75)	0.80 (0.70–1.10)	0.80 (0.70–1.00)	0.50
Lactic acid (mmol/L)-m (p 25–75)	2.30 (1.25–4.58)	1.75 (1.10–2.50)	0.08
ICP (mmHg)-m (p 25–75)	25 (11–30)	15 (14–20)	0.36
CPP (mmHg)-m (p 25–75)	61 (52–70)	68 (57–70)	0.60
APACHE-II score-m (p 25–75)	25 (23–28)	18 (14–22)	<0.001
TAC (mmol/mL)-m (p 25–75)	5.60 (2.45–9.54)	2.35 (1.82–2.86)	<0.001

GCS = Glasgow Coma Scale; n= number; m = median; p 25–75 = percentile 25th–75th; ISS = Injury Severity Score; PaO_2_ = pressure of arterial oxygen; FIO_2_ = fraction inspired oxygen; aPTT = activated partial thromboplastin time; INR = international normalized ratio; ICP = intracranial pressure; CPP = cerebral perfusion pressure; APACHE II = Acute Physiology and Chronic Health Evaluation; TAC = total antioxidant capacity.

**Table 2:** Thirty-day mortality prognostic capability of serum total antioxidant capacity (TAC) levels at day 1, 4, and 8 of trauma brain injury.

	Day 1	Day 4	Day 8
Cut-off of TAC (pg/mL)	>4.32	>2.67	>2.79
Specificity (95% CI)	93% (86–98%)	76% (65–84%)	93% (86–97%)
Sensitivity (95% CI)	59% (41–75%)	82% (57–96%)	69% (38–90%)
Negative predicted value (95% CI)	86% (80–90%)	96% (89–98%)	95% (90–98%)
Positive predicted value (95% CI)	77% (59–88%)	39% (29–49%)	60% (39–77%)
Negative likelihood ratio (95% CI)	0.4 (0.3–0.7)	0.2 (0.1–0.7)	0.3 (0.1–0.7)
Positive likelihood ratio (95% CI)	8.8 (3.9–20.1)	3.4 (2.2–5.2)	10.4 (4.4–24.4)

CI = confidence interval.
